# Academic relationships between Hungarian professors and the Second Vienna Medical School

**DOI:** 10.1007/s10354-021-00840-y

**Published:** 2021-04-14

**Authors:** Béla Szende, Attila Zalatnai

**Affiliations:** Department of Pathology and Experimental Cancer Research, Semmelweis University Budapest, Budapest, Hungary

**Keywords:** ‘Second’ Vienna Medical School, University of Pest/Budapest, Karl Rokitansky, Ignaz Philipp Semmelweis, Mór Kaposi

## Abstract

This article discusses the impact of the ‘second’ Vienna Medical School, hallmarked by Karl Rokitansky, Joseph Skoda and Ferdinand Hebra, on the study and practice of medicine in Hungary. Six medical doctors’ lives and achievements are outlined, who formed a bridge between Vienna and Budapest through their studies and work. Four of them returned to Hungary and promoted the cause of medicine and medical education there. Lajos Arányi (1812–1877) founded in 1844 the Institute of Pathology at the University of Pest. János Balassa (1814–1868) took the Chair of the Surgical Department. Ignaz Philip Semmelweis (1818–1865), the ‘Saviour of Mothers’, received a position at the Department of Obstetrics and Gynaecology in Vienna in 1846. Gustav Scheuthauer (1832–1894) became Arányi’s successor. Each of them continued to keep contact with their tutors in Vienna, especially with Karl Rokitansky, and followed the clinicopathological conception pioneered by the Vienna Medical School regarding diagnostics, treatment and prevention of diseases. Two physicians remained in Vienna: Mór Kaposi (1837–1902), who became known worldwide posthumously due to the connection between Kaposi’s sarcoma and AIDS, was the director of the Department of Dermatology of the Vienna University in 1878. Salomon Stricker (1837–1898) undertook the leadership of the Department of General and Experimental Pathology in 1872.

The ‘second’ Vienna Medical School, hallmarked by Carl Rokitansky, Joseph Skoda and Ferdinand Hebra created an opportunity primarily for citizens of the Habsburg Empire to study and consequently practice medicine at the University of Vienna [1]. The university played a decisive role in the studies of the Hungarian students abroad; it was the most frequently chosen institution. Some of the students shared the time of studies between the medical faculties of Vienna and Pest (founded in 1769). The two universities were interoperable: many students took their first semesters at Pest but the following ones in Vienna, where they received their diploma. Between 1849 and 1890 there were 4621^1^ medical students enrolled at the University of Vienna who were born in the Kingdom of Hungary. A significant proportion of these were students of the medical profession. Some young doctors practiced at various departments of the *Allgemeines Krankenhaus* in Vienna after graduation for 2 to 4, sometimes even up to 10 years (Ignaz Philip Semmelweis, Lajos Arányi, Janos Balassa, Gustav Scheuthauer) or throughout their lives (Mór Kaposi, Salomon Stricker). Lajos Markusovszky, the founder of the *Hungarian Medical Journal* spent 2 years in Vienna at the surgical clinic under supervision of prof. Joseph Wattmann. The best of those who returned to Hungary became professors and heads of various departments (pathology, obstetrics and gynaecology, surgery) at the Faculty of Medicine, Pest University, and were founders of the Pest Medical School. Semmelweis and Kaposi are known worldwide. Each of them continued to keep contact with their tutors in Vienna, especially with Carl Rokitansky, and followed the clinicopathological conception pioneered by the Vienna Medical School regarding diagnostics, treatment and prevention of diseases. Our paper presents the most important achievements of the above-listed Hungarian professors.

Let us start with Lajos Arányi (1812–1877), who after a short period of juristic studies attended the Medical Faculty of the University of Pest in 1832 and got doctor’s degree in 1837. He started practicing medicine but seeking to get closer to the real background of diseases, he went to Vienna where he was fortunate to join the activities going on at Rokitansky’s Department of Pathology at the *Allgemeines Krankenhaus* in 1842, and learned as much as he could about the theory and practice of pathology. In 1844 he returned to Pest and founded the Institute of Pathology of the university with the permission of King Ferdinand V. However, for the first 7 years the Department was run at Arányi’s own expenses. He was the author of the first textbook of pathology in Hungarian language [2], and initiated clinicopathological collaboration in order to improve diagnostic work. The book of autopsy protocols handwritten by Arányi from 1845 are still preserved at the Department of Pathology. He prepared pathological specimens for educational purposes, some of which are still preserved (Fig. 1), together with the catalogue that he compiled. Besides his work at the university, he organised the first ambulance service in Pest and constructed a device supporting respiration in the course of first aid (Fig. 2). Arányi is considered the founding father of Hungarian pathology [3].

**Fig. 1:**
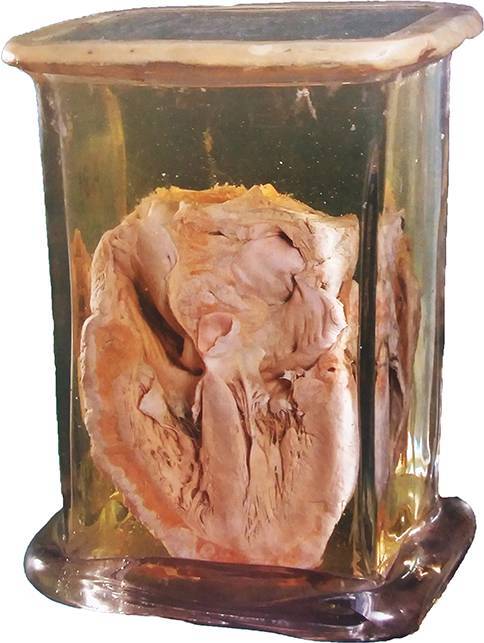
Pathological specimen prepared by L. Arányi (heart). (Reproduced with the permission of the Semmelweis Museum of Medical History, Budapest)

**Fig. 2:**
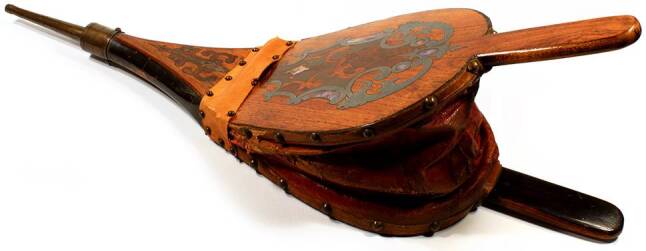
First aid device supporting respiration. (Reproduced with the permission of the Semmelweis Museum of Medical History, Budapest)

Arányi’s good friend, János Balassa (1814–1868), a brilliant surgeon, graduated from the University of Vienna in 1838 and started his professional career at the Department of Surgery of the *Allgemeines Krankenhaus* led by Professor Franz Schuch, first as resident and very soon as deputy head of department. In 1844 he was invited to take the chair of the Surgical Department of the University of Pest [4]. The highlights of Balassa’s professional activity were introducing diethyl ether narcosis as early as 1847 in his Department, and co-founding with L. Markusovszky the *Medical Weekly* (*Orvosi Hetilap*) in 1857, a journal in Hungarian which still exists.^2^ This medical journal was established on the model of the *Wiener Medizinische Wochenschrift*. Although it aimed to facilitate publications in Hungarian, several professors did place their works in *WMW* (Balassa, for example, published 25; Kohn/Kaposi wrote 69 papers). Balassa’s surgical skills were acknowledged by Queen Elisabeth (Sissy), who chose him as physician, surgeon and obstetrician of the Royal Court in Buda. Fulfilling this duty, Balassa helped the Queen to give birth to Princess Maria Valeria in 1868. Balassa’s early death was caused by perforated acute appendicitis.^3^

Ignaz Philip Semmelweis (1818–1865), friend and colleague of Arányi and Balassa, was born in 1818 and grew up in Buda, Hungary. It is not widely known that after graduation from the Medical Faculty in Vienna in 1844, while waiting for a vacancy at the Department of Medicine led by Joseph Skoda, Semmelweis was an external student at Rokitansky’s Department of Pathology at the *Allgemeines Krankenhaus* [7]. By good fortune he was offered the position of assistant professor at the 1st Department of Obstetrics and Gynaecology in 1846, which he accepted. Statistical and autopsy experience led him to discover the infectious origin of puerperal fever and to introduce the method of asepsis, that is, the disinfection of hands and tools using the solution of chloride of lime (Fig. 3). He published his findings under the title *Die Aetiologie, der Begriff und die Prophylaxis des Kindbettfiebers* (The Aetiology, Concept and Prophylaxis of Childbed Fever) in 1861.^4^ Rokitansky, Skoda and Hebra recognised the importance of Semmelweis’s doctrine, but the majority of contemporary obstetricians rejected it. Semmelweis left Vienna in 1850 and became head of the Department of Obstetrics and Gynaecology at the Medical Faculty of the University of Pest, his native city, where he was able to minimalize the ratio of puerperal fever by practicing his doctrine of asepsis. Unfortunately, the discipline of microbiology only started to flourish a short time after his tragic death. He is known and honoured worldwide as the Saviour of Mothers.

**Fig. 3:**
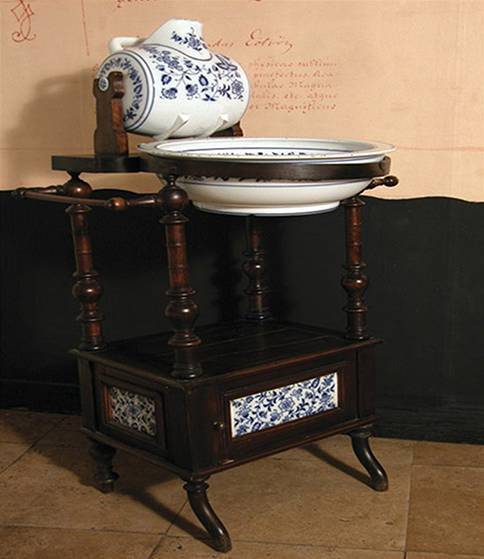
Washbasin for disinfection from the period of Ignaz Semmelweis. (Reproduced with the permission of the Semmelweis Museum of Medical History, Budapest)

Gustav Scheuthauer (1832–1894) graduated together with his classmates Kaposi and Stricker, all Hungarian-born disciples of the great generation of the Second Vienna Medical School. He became Rokitansky’s closest disciple as his second, later first assistant for 10 years. In 1870 he applied successfully for clinical professorship at the Medical Faculty of Pest University, and 4 years later he became successor of the retired Arányi as full professor and director of the Department of Pathology of the same faculty (Fig. 4, [10]). He took special interest in the histopathology of neurogenic tumours, developmental anomalies and pathology of helminthic diseases. Scheuthauer became eponymous for the description of cleidocranial dysplasia, which is known as Scheuthauer–Marie–Stainton syndrome. He first described this syndrome in an article in the *Allgemeine Wiener medizinische Zeitung* in 1871 under the title *Combination rudimentärer Schlüsselbeine mit Anomalien des Schädels beim erwachsenen Menschen* (Combination of rudimentary clavicles with skull anomalies in adult humans).^5^

**Fig. 4:**
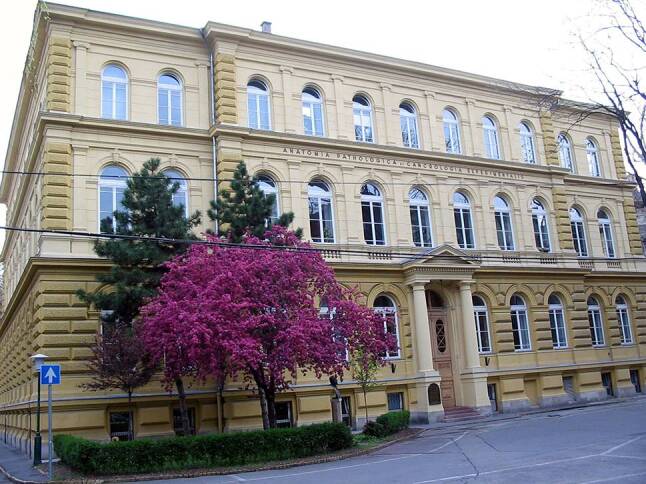
The building of the 1st Department of Pathology of the Semmelweis University, Budapest. Photo: Mária Cserneky 2008 (reproduced with the permission of the copyright holder)

Mór Kaposi (born as M. Kohn; 1837–1902), the “Resurgent Dermatologist”, as two Indian colleagues wrote about him in 2014 [11], has become known worldwide since the connection of Kaposi’s sarcoma and AIDS proved to be evident more than 100 years after his publication on *Idiopathisches multiples Pigmentsarkom der Haut* (Idiopathic multiple pigmentary sarcoma of the skin), named Kaposi’s sarcoma on the suggestion of H. Köbner in 1891 (Fig. 5; [12]). The assistant to Professor Ferdinand Ritter von Hebra was born and grew up in the Hungarian town of Kaposvár. He attended the Medical Faculty of the Vienna University. After his graduation in 1861, he practiced as resident at the *Allgemeines Krankenhaus* and learned syphidology from Ludwig Sigmund. He joined the Department of Dermatology of the Vienna University in 1866 and became full professor and director of the department in 1878, acting until his death. It should be emphasized that Kaposi’s sarcoma was not the only disease he described as the first in the history of medicine. He recognized and published a series of other dermatological alterations as well, such as rhinoscleroma, lichen ruber moniliformis, xeroderma pigmentosum; lichen scrophulosum, Impetigo herpetiformis and systemic lupus erythematosus (SLE).

**Fig. 5:**
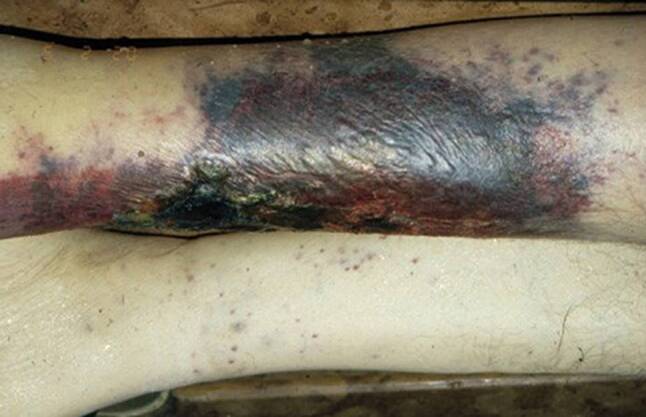
Macroscopic image of Kaposi’s sarcoma [8, p. 57]

**Fig. 6:**
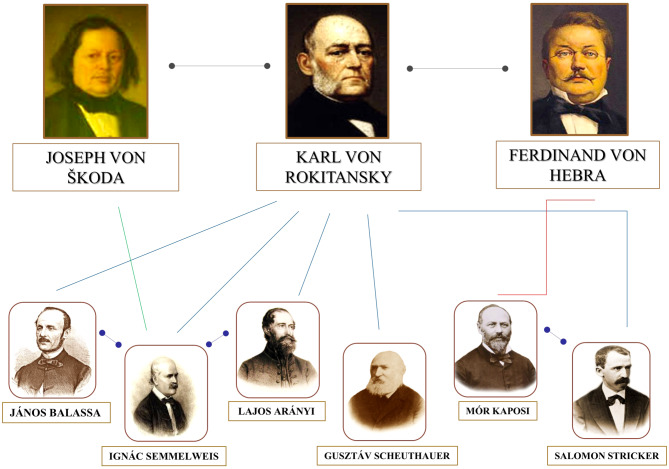
Personal contacts between the ‘second’ Vienna Medical School and medical doctors from Hungary. (Compiled by/copyright Béla Szende)

The last outstanding personality to be presented here is another Hungarian-born professor, Salomon Stricker (1837–1898), who after studies at the Protestant Grammar School in Pest, attended the Medical Faculty of the Vienna University and graduated in 1861. He became the first genuine histopathologist and founder of experimental pathology in Vienna [13]. He was the man who endorsed Kaposi’s findings by performing a histopathological description of the dermatological alterations observed by Kaposi. Stricker became head of the newly established Department of General and Experimental Pathology at the Vienna University on the recommendation of Carl Rokitansky in 1872. He was appointed as supervisor of the clinical departments of the university in 1873 [14].

In Fig. 6. some important personal relationships are presented.

Thanks to our predecessors in the 19th century, despite political and economic difficulties, close scientific and human contacts characterised the relation between the medical schools of Vienna and Pest. I have experienced the same kind of support and sharing of knowledge during the past 50 years from the Viennese pathologists and oncologists, like Professors Holzner, Wrba, Schulte-Hermann, Bursch, Kerjaski and others. It is my wish and hope that this close cooperation continues in the future.

## Footnotes

1 Patyi Gábor: Magyarországi diákok bécsi egyetemeken és főiskolákon 1890–1918. Magyarországi diákok egyetemjárása az újkorban 10. kötet. Bp., 2004. (Hungarian students at the Vienna universities and schools 1890–1918. Hungarian students attending universities at that era. Vol. 10.)

2 An appreciation of his work was published in the same journal in 2003: [5]. For a detailed biography see [6].

3 Balassa’s main works include: Gyakorlati sebészet. Pest, 1844; A hassérvekről. Pest, 1853; Új műtét módozata az orrképzés körül. Pest, 1863; Képző műtétek. Pest, 1867.; Balassa János összegyűjtött kisebb művei. Összeállította Janny Gyula, Bp., 1875. (Practical Surgery, Pest, 1844. About the abdominal hernias, Pest, 1853. Novel surgical method on nasal correction. Pest, 1863. Surgical correction methods, Pest, 1867. Collection of smaller works of Janos Balassa. Edited by Gyula Janny, Bp., 1875.)

4 [8] The English translation of Semmelweis’s study was published as [9].

5 [8, pp. 108, 109].
